# Role of Snf-β in lipid accumulation in the high lipid‐producing fungus *Mucor circinelloides* WJ11

**DOI:** 10.1186/s12934-021-01545-y

**Published:** 2021-02-27

**Authors:** Shaista Nosheen, Tahira Naz, Junhuan Yang, Syed Ammar Hussain, Abu Bakr Ahmad Fazili, Yusuf Nazir, Shaoqi Li, Hassan Mohamed, Wu Yang, Kiren Mustafa, Yuanda Song

**Affiliations:** 1 Colin Ratledge Center for Microbial Lipids, School of Agricultural Engineering and Food Science, Shandong University of Technology, 255000 Zibo, Shandong China; 2 Department of Biology, South Texas Center of Emerging Infectious Diseases (STCEID), University of Texas, 78249 San Antonio, TX USA; 3 Department of Food Sciences, Faculty of Science and Technology, Universiti Kebangsaan Malaysia, 43600 UKM Bangi, Selangor Malaysia; 4 Department of Botany and Microbiology, Faculty of Science, Al-Azhar University, 71524 Assiut, Egypt

**Keywords:** *Mucor circinelloides*, Lipid accumulation, Snf-β, acetyl-CoA carboxylase, Gene expression, Oleaginous fungus

## Abstract

**Background:**

*Mucor circinelloides* WJ11 is a high-lipid producing strain and an excellent producer of γ-linolenic acid (GLA) which is crucial for human health. We have previously identified genes that encode for AMP-activated protein kinase (AMPK) complex in *M. circinelloides* which is an important regulator for lipid accumulation. Comparative transcriptional analysis between the high and low lipid-producing strains of *M. circinelloides* showed a direct correlation in the transcriptional level of AMPK genes with lipid metabolism. Thus, the role of Snf-β, which encodes for β subunit of AMPK complex, in lipid accumulation of the WJ11 strain was evaluated in the present study.

**Results:**

The results showed that lipid content of cell dry weight in Snf-β knockout strain was increased by 32 % (from 19 to 25 %). However, in Snf-β overexpressing strain, lipid content of cell dry weight was decreased about 25 % (from 19 to 14.2 %) compared to the control strain. Total fatty acid analysis revealed that the expression of the Snf-β gene did not significantly affect the fatty acid composition of the strains. However, GLA content in biomass was increased from 2.5 % in control strain to 3.3 % in Snf-β knockout strain due to increased lipid accumulation and decreased to 1.83 % in Snf-β overexpressing strain. AMPK is known to inactivate acetyl-CoA carboxylase (ACC) which catalyzes the rate-limiting step in lipid synthesis. Snf-β manipulation also altered the expression level of the ACC1 gene which may indicate that Snf-β control lipid metabolism by regulating ACC1 gene.

**Conclusions:**

Our results suggested that Snf-β gene plays an important role in regulating lipid accumulation in *M. circinelloides* WJ11. Moreover, it will be interesting to evaluate the potential of other key subunits of AMPK related to lipid metabolism. Better insight can show us the way to manipulate these subunits effectively for upscaling the lipid production. Up to our knowledge, it is the first study to investigate the role of Snf-β in lipid accumulation in *M. circinelloides*.

## Introduction

Microbial lipids have attracted significant research interest during the past few years owing to their benefits and potential to replace conventional sources [1, 2]. Some oleaginous microbes can produce polyunsaturated fatty acids (PUFAs) that are considered essential fatty acids, they must be obtained from external sources as the human body cannot synthesize them [3–5]. Oleaginous fungi are recognized worldwide as potential sources of PUFA with significant commercial value, such as omega 3 and omega 6 PUFAs [6, 7]. The dietary importance of these PUFAs in maintaining human health has been suggested decades ago [8]. *M. circinelloides* is a well-known filamentous fungus, considered to be the microbial factory for the production of useful natural compounds and metabolites [9]. They have the capability of producing a high amount of PUFAs rich in GLA (γ-linolenic acid, 18:3, delta-6,9,12) [8, 10]. GLA is an important omega 6 fatty acid that has a crucial role in upholding human well-being and preventing various health-related issues [11, 12]. For example, GLA has been proposed to be effective in preventing and treating diabetes, cancer, various inflammatory and cardiovascular disorders [13, 14]. The primary GLA production from plant seed has disadvantages to some extent, as the production of GLA from the plant seeds may be affected by region and climate changes, thus GLA rich microbes provide promising alternatives over conventional sources [15]. Since 1980, many studies have been conducted to increase lipid production in *M. circinelloides*. Availability of genomic data and convenient genetic tools have facilitated manipulation of this strain and thus make it an important organism for microbial lipid studies [16–18].

Recently, a new strain of *Mucor*, *M. circinelloides* WJ11 has been isolated and regarded as high lipid producer as it can accumulate up to 36 % of its dry cell weight (DCW) compared to the reference strain CBS 277.49 which could only accumulate 15 % lipid of DCW [19]. To date, many strategies have been developed to increase intracellular lipid content and to reduce the cost of production of valuable fatty acids. One of the known strategies is to increase the supply of pathway precursors by increasing the activity of key enzymes involved in lipid synthesis [20–22]. Previous studies have shown that lipid accumulation is induced under nitrogen limitation while cell growth is decreased and thus affect the overall yield [23, 24]. Therefore, we looked for global regulatory factors associated with lipid metabolism to eventually enhance lipid production without sacrificing the cell growth [25]. We found that AMPK protein kinases may play important role in lipid metabolism as a metabolic regulator. We identified AMPK genes in *M. circinelloides* and did their transcriptional analysis to figure out candidate genes involved in lipid accumulation [26]. AMPKs play significant role in controlling lipid metabolism by regulating the activity of ACC, an essential enzyme for regulating fatty acid synthesis [27, 28]. Our previous study revealed that AMPK exist as a heterotrimeric complex in *M. circinelloides* consisting of three subunits, catalytic α (Snf-α1 and Snf-α2), regulatory β (Snf-β), and γ subunits (Snf-γ1, Snf-γ2, Snf-γ3, Snf-γ4, Snf-γ5, Snf-γ6). Each subunit is further encoded by different distinct genes. All the possible isoform combinations lead to the formation of variety of AMPK complexes. AMPK is regulated by AMP concentration, sometimes a small change in AMP produces a large response. So, AMPK system is activated by rising AMP and falling ATP and once activated upregulate catabolic pathways to promote ATP production and inhibit anabolic pathways to conserve energy.

AMPK could regulate lipid accumulation at transcriptional as well as translational level. The mechanisms by which AMPK regulate transcription of genes is still unclear, possibly by phosphorylation of transcription factors or co transcriptional regulators of genes [29]. However, It has been established that AMPK in its active form phosphorylates and inhibits the activity of ACC, impeding the conversion of acetyl-CoA to malonyl-CoA, an essential precursor of lipid synthesis [30, 31]. Malonyl-CoA also inhibits carnitine palmitoyl transferase (CPT-1), that transport fatty acid into mitochondria for oxidation. So AMPK not only inhibits fatty acid synthesis but also promotes fatty acid degradation [32]. Thus, the deletion of AMPK genes can significantly enhance lipid accumulation.

The previous study conducted by our group has selected the candidate AMPK/SNF1 genes related to lipid accumulation in *M. circinelloides* based on transcriptional profiling [26]. Studies has shown the strong association of AMPK with lipid metabolism including mammals [33], yeast [34] and plants [35] but little is known about the function of AMPK in oleaginous organisms. To date, the role of AMPK in lipid production in *M. circinelloides* using genetic modification has not been studied. So in this study, for the first time, we explored the role of β subunit of AMPK/SNF1 in lipid production in *M. circinelloides* WJ11.

Deletion mutant of β subunit showed enhanced lipid accumulation. We further confirmed our results by overexpressing the same gene in WJ11 strain and the overexpressing mutant showed decreased lipid production. Our results suggested that β subunit acts as a negative regulator of lipid accumulation in *M. circinelloides*.

## Results

### Generation of Snf-β knockout and Snf-β overexpressing strains of M. circinelloides by genetic engineering

**Fig. 1:**
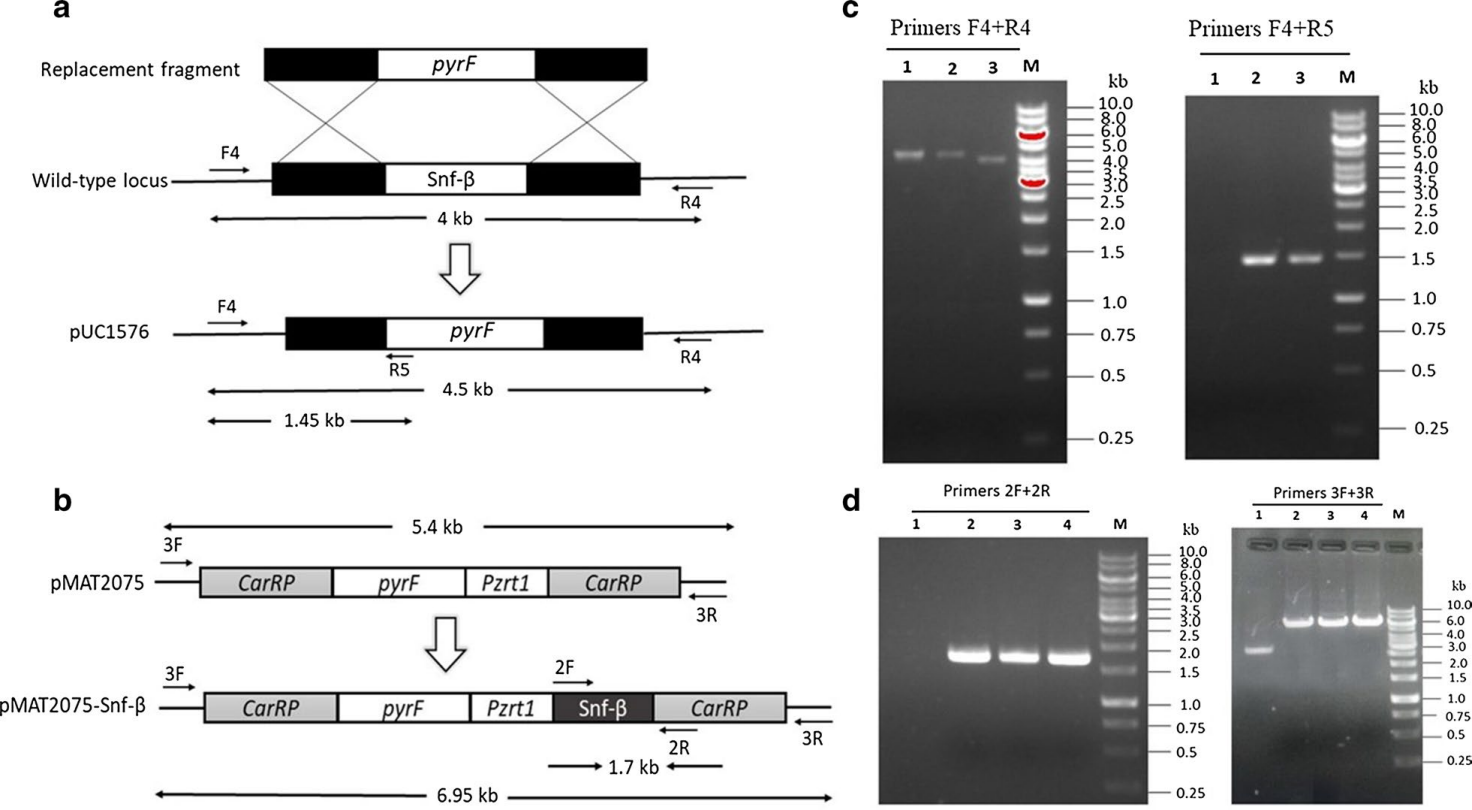
Knockout and overexpression of Snf-β gene **a** Construction of knockout plasmid pUC1576 (lower). Homologous recombination event between wild type locus (middle) with the replacement fragment (upper). F4, R4, R5 are indicating the primers. The arrows showing the expected PCR band size using the indicated primers. **b** Verification of knockout transformants with indicated primers (Left side). Lane M: Marker, 1–2: knockout transformants (MU1576 and MU1577 respectively), 3: Wild type control strain (MU2075) using F4 and R4 primers. Lane M: Marker, 2–3: knockout transformants (MU1576 and MU1577), 1: control strain (MU2075) using F4 and R5 primers (Right side). **c** Plasmid structure of pMAT2075-Snf-β for Snf-β overexpression in *M. circinelloides* is shown. 2F, 2R, 3F and 3R are indicating the primers. **d** PCR amplification of Snf-β gene and plasmid region using primers 2F/2R. Lane M: Marker, Lane 2, 3, 4 showing the presence of Snf-β gene in overexpressing transformants Mc3075, Mc3076, Mc3077 respectively and Lane 1 representing the control strain. Verification of the overexpressing strain by amplification of the whole *carRP* locus with the primers 3F/3R. Lane M: Marker, Lane 2, 3, 4 showing the presence of Snf-β gene including whole *carRP* locus in overexpressing transformants Mc3075, Mc3076, Mc3077 respectively and Lane 1 representing *carRP* locus in Mc2075 strain without Snf-β gene (carrying the empty vector pMAT2075). Sizes in the kb of the relevant fragment are indicated

Based on protein sequence alignment, one gene (Scaffold00011.47) that codes for β subunit of SNF1/AMPK complex was found, and named as Snf-β [26].To determine the role of Snf-β in fatty acid accumulation, both knockout mutants and overexpressing strains of this gene were generated. Gene replacement strategy was used to generate knockout mutants. Knockout vector pUC1576 was designed containing the *pyrF* gene as a selective marker, flanked by the adjacent sequences of the Snf-β gene (Fig. 1a). The designed knockout vector was digested with *Sma*I and transformed in M65 strain to obtain positive transformants (indicating Snf-β gene region in transformants replaced by *pyrF* marker region). Initially, *M. circinelloides* transformants are heterokaryons due to the presence of several nuclei in the protoplasts, but when grown in a selective medium for several vegetative cycles, homokaryotic transformants were obtained [36]. Thus, the transformation of M65 strain produced two independent homokaryotic transformants (MU1576 and MU1577).

The disruption of the gene in transformants MU1576 and MU1577 was confirmed by PCR analysis (Fig. 1b). Primer pair (F4 and R4) was designed that amplified the Snf-β gene and surrounding sequences and amplification produced the expected 4-kb fragment in the control strain, whereas in the knockout positive transformants, amplification produced 4.5-kb fragment corresponding to the disrupted allele. The presence of a single required band of 4.5-kb indicated that transformants are homokaryotic for the disruption. Disruption of the gene was further confirmed with a second PCR amplification using primer pair (F4 and R5) that could amplify only the disrupted locus as F4 hybridized with the upstream sequences of Snf-β gene and R5 with the *pyrF* gene. Amplification results produced the expected 1.45-kb band on the gel that confirmed Snf-β disruption in transformants while no band was observed for control strain (Fig. 1b). Thus, PCR amplification results confirmed the disruption of the Snf-β gene in the genome of the recombinant fungus. CDW and lipid analysis was carried out for both transformants and both produced comparable results (Additional file 1: Fig. S1) thus only one strain MU1576 was selected for further evaluation.

To overexpress the Snf-β gene, a plasmid named pMAT2075-Snf-β was generated that contains the target gene region under the control of the strong zrt1 promoter of *M. circinelloides* [37]. pMAT2075-Snf-β and the empty plasmids pMAT2075 was transformed into strain M65 (Fig. 1c). Three independent Snf-β overexpressing transformants, named Mc3075, Mc3076, and Mc3077 were selected. The overexpression of the Snf-β gene in transformants was confirmed by PCR analysis. Amplification was carried out using a primer pair (2F and 2R) that amplified the target gene and 124 bp sequences of the plasmid pMAT1552. As expected, a 1.7-kb fragment was amplified in overexpressed transformants, but no fragment was amplified in the control strain. (Fig. 1d). Thus, PCR amplification results confirmed that the target gene has been integrated into the genome of the transformants. Verification of the overexpressing strain was also done by amplification of the whole *carRP* locus with the primers 3F/3R. Overexpressing strain harboring the plasmid pMAT2075-Snf-β produced the 6.95 kb fragment while strain carrying empty pMAT2075 plasmid produced 5.4 kb fragment on the gel (Fig. 1d). The presence of single band indicated homokaryosis for the overexpressing strain.

Overexpressing strains were grown in 150 mL K & R medium for 3 to 4 days in 500 ml baffled flasks. Since cell dry weight (CDW) and lipid of overexpressing strains (Mc3075, Mc3076 and Mc3077) were comparable (Additional file 1: Fig. S1) thus only one strain Mc3075 was selected for further evaluation. CDW and lipid content of wild type strain (MU2075) and strain with empty pMAT2075 plasmid (Mc2075) were analyzed and both produced comparable results. So, for ease of understanding and interpretation of results, mutants were compared to one wild type control (MU2075) in this study.

### The expression level of Snf-β gene in Snf-β knockout and Snf-β overexpressing strains

**Fig. 2:**
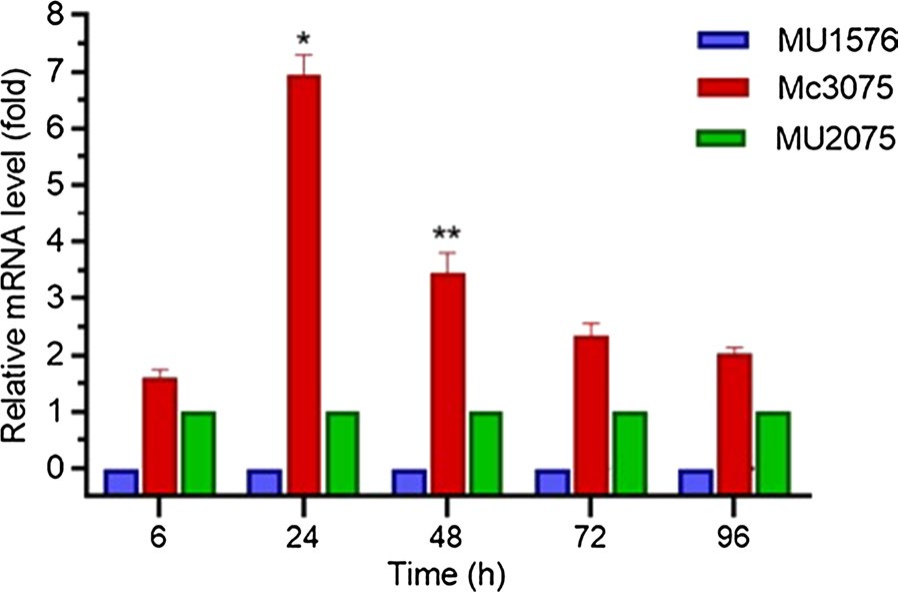
Determination of expression levels of the Snf-β gene by RT-qPCR in the knockout strain MU1576, overexpressing strain Mc3075 and the control strain MU2075. Error bars represent standard deviations (*n* = 3), and asterisks indicate significant differences: *P < 0.05 and **P < 0.01. The least significant difference (LSD) values of Mc3075 and MU2075 were 0.6910 and 0.1009, respectively

RT-qPCR was carried out to analyze the mRNA levels of the Snf-β gene in transformants (MU1576 and Mc3075) and wild type control strain (MU2075) at 6, 24, 48, 72, and 96 h of growth using the primers Snf-β-F/ Snf-β-R (Additional file 1: Table S1). In the Snf-β knockout strain, the mRNA level of Snf-β gene was undetectable throughout the culture time and thus confirmed the gene disruption in the knockout transformant. However, in overexpressing strain, mRNA level of Snf-β gene was significantly increased as compared to the control. The expression level was quickly increased at the start of fermentation and reached 6.4 fold at 24 h but showed a decreasing trend afterward but maintained at an elevated level throughout the fermentation confirming that the Snf-β gene was overexpressed in the corresponding recombinant strains (Fig. 2).

### Cell growth and lipid accumulation in Snf-β knockout and Snf-β overexpressing strains

**Fig. 3:**
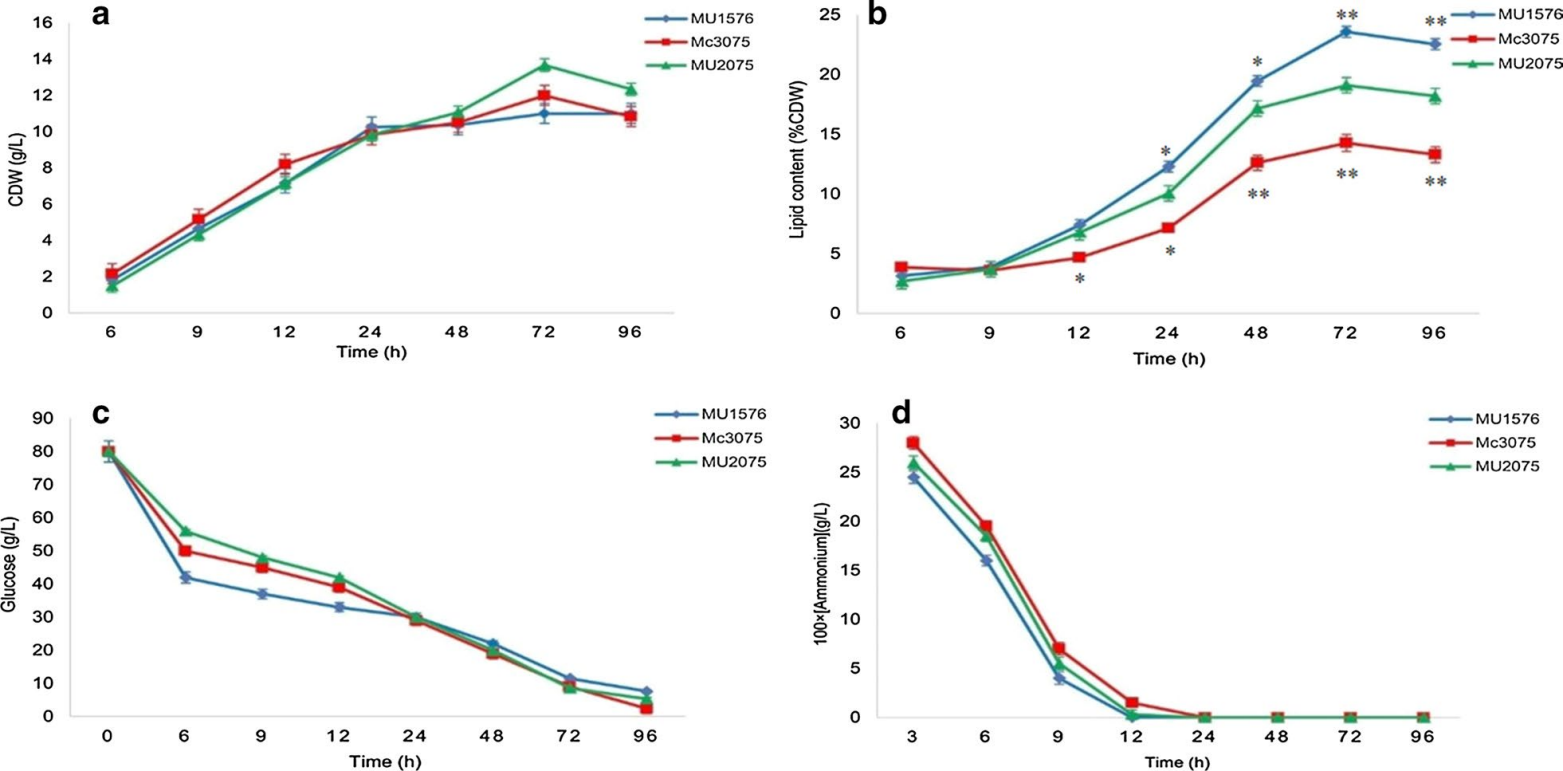
Cell growth and lipid content analysis of Transformants and control strains at indicated time intervals **a** cell dry weight (CDW), **b** lipid content, **c** glucose concentration **d** ammonium concentration were measured. Error bars represent standard deviations (*n* = 3), and asterisks indicate significant differences: *P < 0.05 and **P < 0.01. The LSD values of MU1576, Mc3075, and MU2075 were 0.3867, 0.6809, and 0.3711, respectively

CDW, glucose and ammonium concentration in the culture media and lipid accumulation in Snf-β knockout and Snf-β overexpressing strains were analyzed and compared to the control strain as shown in Fig. 3.

All three strains showed a similar growth pattern as the control strain. CDW increased rapidly during the first 24 h but slowed down after nitrogen depletion and growth ceased after 72 h. The rate of glucose and ammonium consumption was similar in these three strains. Glucose concentration was enough throughout the fermentation. During 12–24 h nitrogen was depleted and the rate of lipid accumulation was rapidly increased after nitrogen exhaustion. Maximum lipid content was achieved at 72 h. Manipulation of the Snf-β gene significantly influenced the lipid accumulation in *M. circinelloides.* Deletion of Snf-β gene enhanced the lipid accumulation in *M. circinelloides* WJ11. Lipid content in Snf-β knockout strain MU1576 was increased by 32 % compared with the control strain MU2075 (from 19 % in the control strain to 25 % in the recombinant strain). However, in Snf-β overexpressing strain Mc3075, lipid content was decreased to 14.2 %, a decrease of 25 % compared to the control strain. Total fatty acid profile analysis revealed that the expression of the Snf-β gene did not significantly affect the fatty acid composition of the strain (Table 1). However, GLA content in biomass was increased from 2.5 % in control strain to 3.3 % in Snf-β knockout strain due to increased lipid accumulation. On the contrary, GLA content in biomass was decreased to 1.83 % in Snf-β overexpressing strain.

**Table 1:** Fatty acid composition in Snf-β knockout and Snf-β overexpressing strain

Strains	Time (h)	Fatty acid composition (relative %, w/w)^a^				
		16:0	18:0	18:1	18:2(LA)	18:3 (GLA)
MU2075	12	19.64 ± 0.24	5.16 ± 0.07	35.68 ± 0.19	16.03 ± 0.09	21.30 ± 0.28
	24	20.09 ± 0.15	6.21 ± 0.27	35.99 ± 0.28	15.99 ± 0.92	15.83 ± 0.71
	48	22.14 ± 0.32	7.74 ± 0.22	37.22 ± 0.51	13.78 ± 0.81	13.80 ± 0.62
	72	23.13 ± 0.08	6.63 ± 0.61	38.0 ± 0.19	14.10 ± 0.13	13.67 ± 0.37
	96	22.49 ± 0.11	5.55 ± 0.31	37.09 ± 0.31	13.61 ± 0.07	12.92 ± 0.09
MU1576	12	19.53 ± 1.21	7.84 ± 1.11	34.88 ± 0.19	15.01 ± 0.61	22.23 ± 0.12
	24	21.52 ± 0.33	6.12 ± 0.45	34.08 ± 1.11	14.95 ± 0.26	15.95 ± 0.04
	48	23.79 ± 0.42	6.83 ± 0.71	36.54 ± 0.09	14.43 ± 0.31	14.18 ± 0.33
	72	24.98 ± 0.80	5.08 ± 0.23	37.33 ± 1.07	14.08 ± 0.21	13.97 ± 0.74
	96	22.07 ± 0.07	5.14 ± 0.04	37.01 ± 0.24	13.49 ± 0.03	14.32 ± 0.05
Mc3075	12	19.98 ± 0.34	5.17 ± 0.26	35.88 ± 0.09	15.51 ± 0.08	19.52 ± 0.76
	24	21.29 ± 0.23	5.22 ± 0.13	36.75 ± 0.80	14.94 ± 0.41	15.79 ± 0.05
	48	23.51 ± 0.12	5.36 ± 1.24	37.20 ± 0.13	14.06 ± 1.24	13.26 ± 0.13
	72	23.83 ± 1.32	4.97 ± 0.09	37.55 ± 0.25	13.43 ± 1.12	13.11 ± 0.34
	96	24.25 ± 0.54	4.48 ± 1.11	38.13 ± 0.51	12.76 ± 0.72	13.09 ± 0.11

^a^The fatty acid composition displayed at different point times. The values are means ± standard deviations of three independent experiments

## Expression levels of ACC1 and ACC2 genes in the transformants

**Fig. 4:**
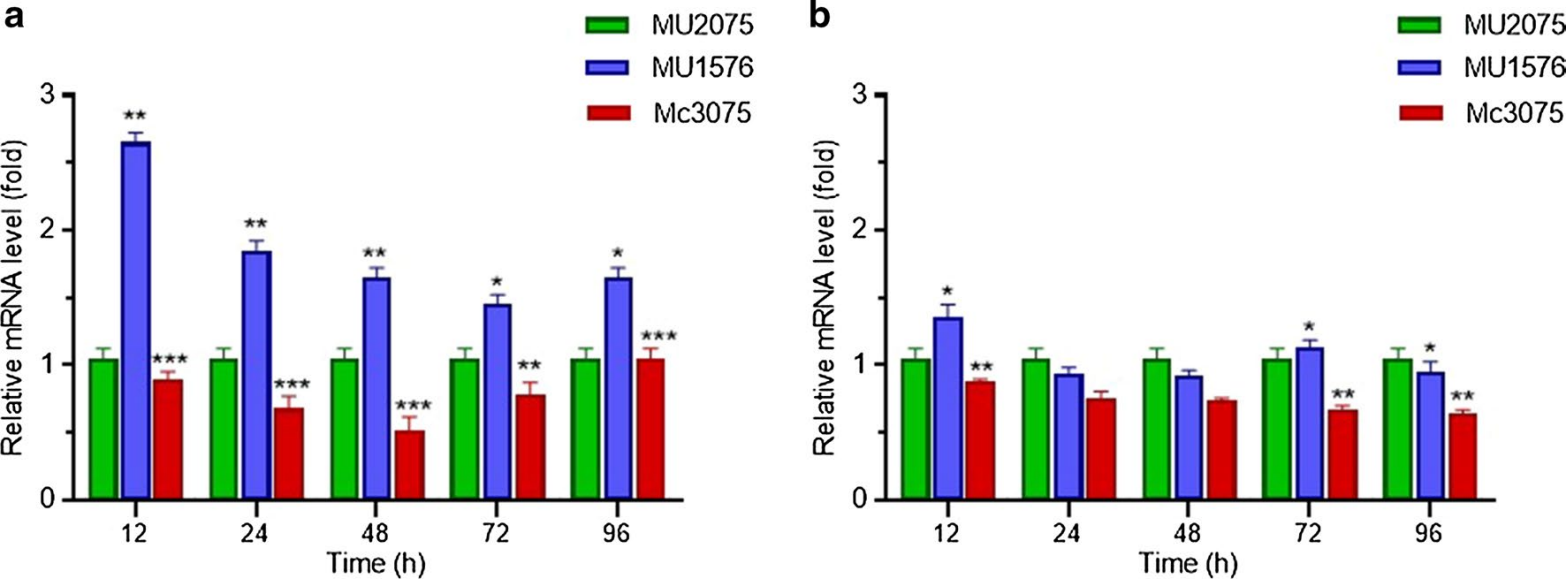
Expression of ACC1 and ACC2 genes in the knockout strain MU1576, overexpressing strain Mc3075 and control strain MU2075 by qRT-PCR. **a** Expression level of ACC1 gene and LSD values of MU1576 and Mc3075 were 0.1630 and 0.2220, respectively. **b** Expression level of ACC2 gene and LSD values of MU1576 and Mc3075 were 0.2260 and 0.0912, respectively. Error bars represent standard deviations (*n* = 3), and asterisks indicate significant differences: *P < 0.05, **P < 0.01; ***P < 0.001

ACC plays an essential role in controlling lipid synthesis and degradation [32, 38]. It is known to be highly regulated by AMPK/SNF1 in eukaryotes. Mammals, fungi, and yeasts contain two isoforms of the ACC, both encoded by distinct genes [39]. To determine the effect of Snf-β manipulation on the expression of the ACC gene, mRNA levels of ACC1 and ACC2 were analyzed in the transformants and compared to the control strain. qRT-PCR analysis showed that mRNA levels of ACC1 in Snf-β knockout strain were higher than that of control strain throughout the fermentation, but expression level gradually decreased with the time from approximately 2.7 fold increase at 12 h to 1.5 fold increase at 72 h compared to control. In Snf-β overexpressing strain, mRNA levels of ACC1 were decreased as compared to control (Fig. 4). The results indicate that the manipulation of Snf-β gene significantly influenced ACC1 expression levels. However, the qRT-PCR analysis has not shown much significant change in the ACC2 expression level in the transformants. This suggested that β subunit of AMPK/SNF1 might control lipid metabolism by regulation of the ACC1 gene.

## Discussion

Several studies have shown the regulatory role of AMPK/SNF1 in lipid metabolism [27, 38]. Manipulation of such regulators may have a high potential to enhance lipid accumulation. In our previous study, we identified AMPK/SNF1 subunits in the genomes of high and low lipid producing strains of *M. circinelloides* and performed their transcriptional analysis. Based on that data, candidate subunits that might be more involved in lipid metabolism were selected. In this study, we determined the role of Snf-β gene, coding for β subunit of AMPK/SNF1, in lipid accumulation in high lipid producing strain *M. circinelloides* WJ11. Our study has shown that manipulation of Snf-β gene significantly affects lipid production. Knockout of β subunit enhanced the lipid production and improved the GLA content as well. These results indicated that β subunit negatively regulates lipid accumulation in WJ11. We confirmed our findings by overexpressing the same gene in *M. circinelloides* WJ11. Overexpression of Snf-β gene significantly decreased the lipid content.

Growth patterns and biomass (CDW) of transformants and control strain were found to be similar. This indicates that Snf-β gene did not significantly affect the growth of *M. circinelloides* WJ11. The pattern of nitrogen and glucose consumption was almost similar in all the strains. Generally, nitrogen limitation in the medium initiate lipid accumulation in oleaginous microbes and lipid production significantly increases under nitrogen depletion [40]. A similar trend was observed in this study. Before nitrogen depletion, lipid production was at a minimal level and there was no considerable difference in lipid content among strains. However, after nitrogen depletion, lipid production was rapidly increased in the strains. Our study demonstrated that manipulation of Snf-β gene had a pronounced effect on lipid accumulation in *M. circinelloides* WJ11. Knockout of Snf-β gene enhanced the lipid accumulation by 32 % as compared to the control strain. A previous study on *Yarrowia lipolytica* also demonstrated that deletion of a β subunit increased the TFA content to 27 % compared to the control strain [34]. A possible explanation for increased lipid production is that deletion of β subunit could reduce the number of active AMPK complexes resulting decrease AMPK levels and consequently affecting the expression and activity of ACC gene. Thus, the conversion of acetyl-CoA to the first building block for fatty acid synthesis, malonyl-CoA will be enhanced that ultimately leads to more lipid production. Previous research in yeast and fungi demonstrated that increased ACC activity results in increased lipid accumulation. Heterologous expression of ACC1 gene of *Mucor rouxii* enhanced fatty acid content by 40 % in non-oleaginous yeast, *Hansenula polymorpha* [39]. Similarly, Wang et al. showed that overexpression of the ACC1 gene from oleaginous yeast *Lipomyces starkeyi* increased the lipid content up to 9.6 % in *S. cerevisiae* [41]. Fatty acid profile analysis showed that expression of Snf-β gene did not significantly affect the fatty acid composition of the strains. It should be noted that in the present study, we manipulated one gene in a strain however co-expression of key genes/subunits can be beneficial to further improve lipid production and to enhance the levels of desired fatty acids.

We confirmed our results by overexpressing the same gene in *M. circinelloides*. In Snf-β overexpressing strain, lipid content was decreased by 25 % compared to the control strain. To determine the effect of the Snf-β gene on ACC expression levels, the mRNA levels of two isoforms of ACC (ACC1 and ACC2) were analyzed by qRT-PCR. Knockout of Snf-β gene upregulated the expression level of ACC1 but change in ACC2 expression level was not much significant. This indicates that Snf-β gene may control lipid accumulation by regulating ACC1 gene. However, it is noteworthy to mention that culture conditions used in our study may not favor the optimal activity of AMPK subunits as they are fully active under stress conditions such as glucose depletion [42, 43] but in our culture conditions, glucose was enough throughout the fermentation. Moreover, subunits may also regulate lipid accumulation by post-transcriptional or post-translational regulatory processes [44, 45]. So, detail studies are required to identify the downstream targets of AMPK subunits and to understand the molecular mechanism of such regulation. Moreover, it will be interesting to test the genetic modification of other subunits that may play a major role in regulating lipid accumulation. Seip et al. demonstrated that the deletion of α subunit increased the lipid production up to 50 % higher than that of the control strain. They also demonstrated that knockout of α and γ subunit exert more prominent effect on lipid increase in growth as well as oleaginous conditions as compare to β subunit deletion [34]. There are several evidence supporting the fact that genetic manipulation of the key subunits is also feasible in engineered microbes to further increase the content of desired fatty acids. A study has shown that α deletion in engineered eicosapentaenoic acid (EPA) producing *Y. lipolytica* strain increased the EPA production by 52 % compared to the control strain. The application of this trait is not limited to EPA production but can be applied to many other industrial settings to produce valuable lipid-derived metabolites.[34].

## Conclusions

This is the first study to determine the role of β subunit of AMPK/SNF1 in lipid accumulation in *M. circinelloides* WJ11. Analysis of knockout and overexpressing strains for the Snf-β gene revealed that β subunit may not be the sole rate-limiting enzyme but as a negative regulator for the lipid accumulation. Knockout of Snf-β gene improved the lipid content by 32 % compared to the control strain. This study proposed that Snf-β may control lipid accumulation by regulating the ACC1 gene and thus increasing the pool of malonyl-CoA precursors for fatty acid synthesis. However further research is required to evaluate the role of other subunits of AMPK/SNF1 in lipid accumulation and to determine their respective downstream targets. Better insight can show us the way to manipulate these subunits effectively to increase lipids production.

## Material and method

### Strains and culture conditions

*M. circinelloides* WJ11 (CCTCC No. M 2014424) was used as the source of the Snf-β gene, β subunit of AMPK/SNF1. *M. circinelloides* M65, uracil auxotroph of *M. circinelloides* WJ11 was used as recipient strain in transformation experiments to knockout and overexpress the Snf-β gene. Cultures were grown at 26 °C in YPG or MMC medium [46, 47]. Media were supplemented with uridine (200 µg mL^− 1^) when required. The pH was adjusted to 3 and 4.5 for colonial and mycelial growth, respectively. Snf-β knockout (MU1576), Snf-β overexpressing (Mc3075), wild type strain (MU2075) as the control and strain harboring empty pMAT2075 plasmid (Mc2075) were initially cultivated by inoculating 100 µL of spore suspension (approximately 10^7^spores/mL) in 500 mL baffled flasks containing 150 mL Kendrick and Ratledge (K & R) medium [48] for 24 h with 150 rpm shaking at 28 °C and then inoculated at 10 %(v/v) as a seed culture into a 2-L fermenter (BioFlo/CelliGen 115, New Brunswick Scientific, Edison, NJ, USA) containing 1.5 L modified K & R medium containing 80 g glucose/L. Fermenters were held at 28 °C stirred at 700 rpm with aeration of 0.5 volume of air per volume of fermenter per minute (v/v min^− 1^), and pH was maintained at 6 by automatic addition of 2 M NaOH or 2 M HCl. *Escherichia coli* DH5α, grown in LB medium with shaking at 220 rpm at 37 °C was used for all cloning experiments [49].

### Plasmids construction

#### Snf-β knockout plasmid

To knockout the Snf-β gene, we assembled a knockout (KO) cassette comprising the *pyrF* marker flanked by ∼1 kb of 5′ and 3′ up- and downstream regions of the Snf-β gene for gene disruption. To generate this cassette, first we amplified the Snf-β gene with ∼1 kb of 5′ and 3′ up- and downstream regions of the Snf-β gene by PCR using primers Snf β-F1- *Sma*I and Snf β-R1- *Sma*I (Additional file 1: Table S1). Primers were designed to introduce *Sma*I cutting side, at both ends of amplified fragments for later construct release. The obtained 3.8-kb PCR fragment was cloned into the PUC-18 vector to give plasmid pUC18-Snf β plasmid. Then PCR was performed using primers F2- *Nhe*I and R2- *SnaB*I to amplify the plasmid thus coding gene fragment was excluded from the plasmid. Meanwhile, the *pyrF* gene was amplified using specific primer pair (F3- *SnaB*I and R3- *Nhe*I). Amplified *pyrF* fragment and reverse PCR fragment was then rejoined to generate KO plasmid (pUC1576). In this way, gene region was replaced by *PyrF* marker in plasmid. knockout (KO) cassette (comprising the *pyrF* marker flanked by ∼1 kb of 5′ and 3′ up and downstream regions of the Snf-β gene) was released from plasmid pUC1576 by *Sma*I and introduced into M65 protoplasts by electroporation-mediated transformation method.

#### Snf-β overexpressing plasmid

The plasmid, pMAT2075 was used for the construction of an Snf-β-overexpressing plasmid. It contains the *M. circinelloides* WJ11 *pyrF* genes surrounded by1 kb up and downstream *carRP* sequences to allow its chromosomal integration by homologous recombination [37]. Isolation of Snf-β gene was carried out by PCR amplification from the genome of *M. circinelloides* WJ11 using the primers Snf-β-1F-*Xho*I/ Snf-β-1R-*Xho*I (Additional file 1: Table S1). The 30 bp homologous sequences of both sides of *Xho*I restriction site in pMAT2075 were also included in these primers. After digestion with *Xho*I restriction endonuclease, the PCR fragment was cloned into plasmid pMAT2075 to generate plasmid pMAT2075-Snf-β (One-step cloning kit, Takara). The recombinant plasmid was transformed into *E. coli* competent cells for its propagation. The plasmids extracted from these bacteria were checked by PCR analysis and DNA sequencing was carried out to confirm the gene sequence. Plasmid pMAT2075-Snf-β was digested with *Sma*I to release the overexpression construct and the released construct was transformed to M65 protoplast. Integration in *carRP* locus produced white colonies, which were easily distinguishable from yellow transformants that did not integrate the gene. The selection of albino colonies was performed out by the method of Rodríguez-Frómeta et al. [50].

#### Transformation method

Transformation was done by electroporation-mediated transformation using the method of A. Gutiérrez et al. [51] with minor modifications. Fresh spores of uracil auxotroph strain, M65 of *M. circinelloides* WJ11 was grown on YPG media supplemented with uridine and maintained at 4 °C overnight without shaking and then incubated at 28 °C at 200 rpm for spore germination for 3–4 h. Germinated spores were washed with ice-cold PS buffer. To digest the cell wall, germinated spores were resuspended in 5 ml of PS buffer treated with 0.3 µl chitosanase RD and 1 mg/ml lysing enzyme. The prepared protoplasts were then suspended in 800 µl of 0.5 M sorbitol to use for electroporation of eight different transformation experiments.

For electroporation, 3–5 µg of linear plasmid in a volume of 10 µl were added to the 100 µl of protoplasts and an electrical pulse was applied using the following conditions: field strength of 0.8 kV, capacitance of 25 µF, and constant resistance of 400 Ω. Immediately after electrical pulse, 1 ml of cold YPG medium was added and incubated for 1 h at 26 °C at 100 rpm. Protoplasts were recovered by centrifugation, resuspended in 500 µl of YNB containing 0.5 M sorbitol, and spread on selective medium plates (MMC plus 0.5 M sorbitol) and incubated in the dark at 28 °C for 3 to 4 days.

### Genomic DNA preparation

For DNA extraction, all strains were grown in K &R medium for 3 days at 28 °C with continuous shaking at 150 rpm. Subsequently, the mycelium was collected by vacuum filtration and then washed thrice with distilled water. Genomic DNA extraction was done using the DNA quick Plant System Kit (Tiangen Biotech Co., Ltd., Beijing, China) according to the manufacturer’s instructions.

### Gene expression analysis by reverse transcription‐quantitative PCR (RT-qPCR)

Control and recombinant strains were grown in a 2 L fermenter containing 1.5 L modified K and R medium. To carry out RT-qPCR analysis, mycelium was harvested at 6, 12, 24, 48, 72 and 96 h. Total RNA was isolated with Trizol after grinding the mycelium under liquid N_2_. The extracted RNA was reverse transcribed using the PrimeScriptTM RT reagent kit (Takara) according to the manufacturer’s instructions. The RT-qPCR analysis was carried out on LightCycler 96 Instrument (Roche Diagnostics GmbH, Switzerland) with FastStart Universal SYBR Green Master mix (ROX) Supermix (Roche) according to the manufacturer’s instructions. Quantification of data was done by the method of 2^−ΔΔCt^ using the 18S rRNA gene of *M. circinelloides* as a housekeeping gene. The results were expressed as relative expression levels.

#### Measurement of glucose and nitrogen concentration in culture medium

The glucose concentration in the culture medium was measured using a glucose oxidase Perid-test kit according to the protocol supplied by the manufacturer (Shanghai Rongsheng Biotech Co., Ltd.). Ammonium concentration was determined by the indophenol method [52].

### Analysis of CDW and lipid accumulation

Biomass samples were harvested by filtration and washed thrice with distilled water. Collected mycelia were frozen overnight at − 80 °C, freeze-dried and the CDW was determined gravimetrically. The lipid extraction was carried out using the method of [53] with minor modifications and the analysis was conducted using the procedure reported in our previous work. Approximately 20 mg lyophilized biomass was mixed with chloroform/methanol (2:1, v/v), and pentadecanoic acid (15:0, Sigma) was added as an internal standard. 10 % (v/v) methanolic HCl was added to the extracted lipid for 3 h at 60 °C. The resultant fatty acid methyl esters (FAMEs), extracted with n-hexane, were analyzed by GC equipped with a 30 m ×0.32 mm DB-Waxetr column with 0.25 µm film thickness (Shimadzu Co., Ltd., Kyoto, Japan). The program used for GC machine was as follow: 120 °C for 3 min, then ramp from 120 °C to 200 °C at the speed of 5 °C/min, then ramp to 220 °C at 4 °C/min and hold for 2 min.

#### Statistical analysis

All the experiments were conducted in triplicate and statistical analysis was carried out by one-way ANOVA followed by Tukey’s multiple comparison tests using GraphPad Prism (version 7, San Diego, CA), and *P* < 0.05 was observed to establish significant differences.

## Supplementary Information

**Additional file 1: Fig. S1.** Cell growth and lipid content analysis of transformants and control strains at 96 h: a) cell dry weight (CDW) of wild type control (MU2075) and knockout transformant (MU1576 and MU1577), b) lipid content of MU2075, MU1576 and MU1577, c) cell dry weight (CDW) of wild type control (MU2075), strain with empty pMAT2075 plasmid (Mc2075) and overexpressing transformant (Mc3075, Mc3076, Mc3077) d) lipid content of MU2075, Mc2075 Mc3075, Mc3076, Mc3077. Error bars represent standard deviations (n = 3). **Table S1.** Primer sequences used in this study.

## Data Availability

All data generated or analyzed during this study are included in this published article [and its additional file].
