# Supplementary material for: Role of Snf-β in lipid accumulation in the high lipid‐producing fungus Mucor circinelloides WJ11
Source: Microb Cell Fact. 2021 Feb 27;20:52. doi: 10.1186/s12934-021-01545-y (PMC7916304; doi:10.1186/s12934-021-01545-y)
Supplement: Supplementary file 1 — Additional file 1: Fig. S1. Cell growth and lipid content analysis of transformants and control strains at 96 h: a) cell dry weight (CDW) of wild type control (MU2075) and knockout transformant (MU1576 and MU1577), b) lipid content of MU2075, MU1576 and MU1577, c) cell dry weight (CDW) of wild type control (MU2075), strain with empty pMAT2075 plasmid (Mc2075) and overexpressing transformant (Mc3075, Mc3076, Mc3077) d) lipid content of MU2075, Mc2075 Mc3075, Mc3076, Mc3077. Error bars represent standard deviations (n = 3). Table S1. Primer sequences used in this study. [file 12934_2021_1545_MOESM1_ESM.docx]

Additional File 1

**Role of Snf-β in lipid accumulation in the high lipid-producing fungus *Mucor circinelloides* WJ11**

Shaista Nosheen^1†^, Tahira Naz^1†^, Junhuan Yang^1^, Syed Ammar Hussain^1,2^, Abu Bakr Ahmad Fazili^1^, Yusuf Nazir^1,3^, Hassan Mohamed^1,4^, Shaoqi Li^1^, Wu Yang^1^, Kiren Mustafa^1^, Yuanda Song^1^ ⃰.

^1^ Colin Ratledge Center for Microbial Lipids, School of Agricultural Engineering and Food Science, Shandong University of Technology, Zibo 255000, Shandong, China

^2^ Department of Biology, South Texas Center of Emerging Infectious Diseases (STCEID), University of Texas, San Antonio, TX 78249, USA

^3^ Department of Food Sciences, Faculty of Science and Technology, Universiti Kebangsaan Malaysia, 43600 UKM Bangi, Selangor, Malaysia

^4^ Department of Botany and Microbiology, Faculty of Science, Al-Azhar University, Assiut 71524, Egypt

Email address and telephone number of the corresponding author:

Yuanda Song: ysong@sdut.edu.cn

+86 13964463099

†Both authors contributed equally to this work


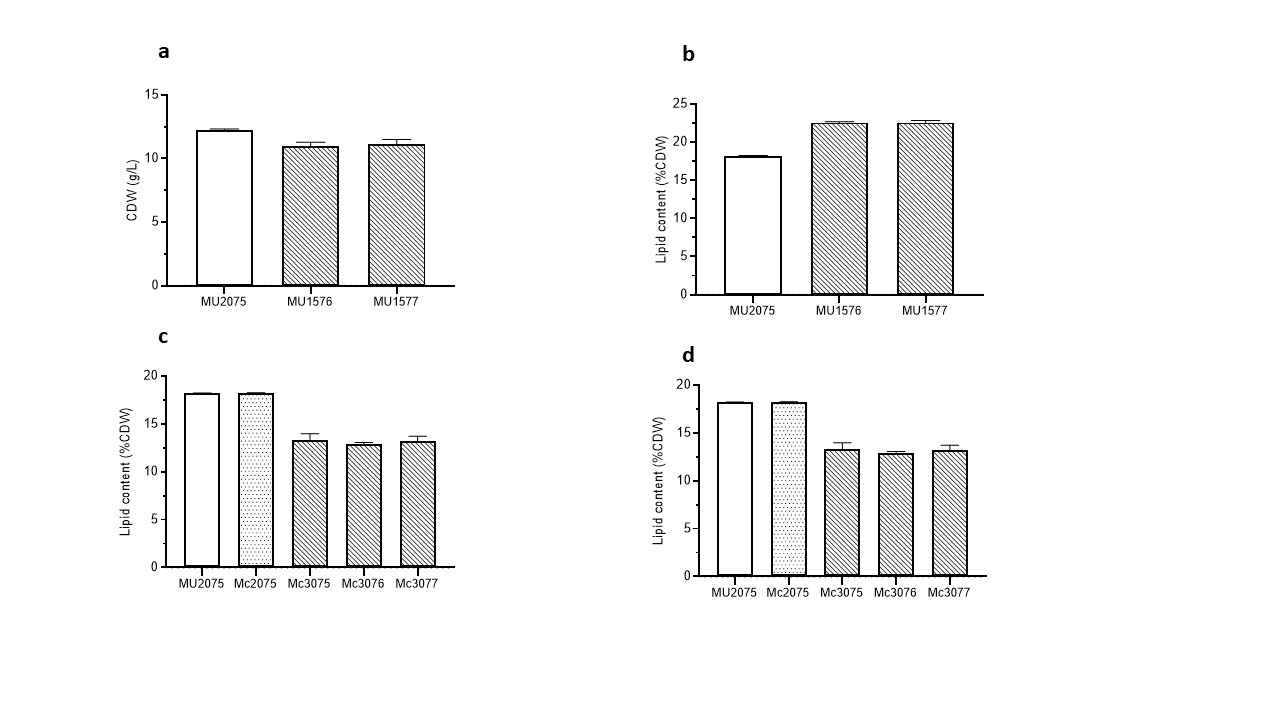


**Fig S1.** Cell growth and lipid content analysis of transformants and control strains at 96 h: a) cell dry weight (CDW) of wild type control (MU2075) and knockout transformant (MU1576 and MU1577), b) lipid content of MU2075, MU1576 and MU1577, c) cell dry weight (CDW) of wild type control (MU2075), strain with empty pMAT2075 plasmid (Mc2075) and overexpressing transformant (Mc3075, Mc3076, Mc3077) d) lipid content of MU2075, Mc2075 Mc3075, Mc3076, Mc3077. Error bars represent standard deviations (*n* = 3)

**Table S1.** Primer sequences used in this study.

| Primer name | Primer sequence (5’-3’) |
| --- | --- |
| Snf β-F1- *Sma*I | CGATGCCCGGGCTGACAAGCATGCGATGAGGATACTGTGAAG |
| Snf β-R1- *Sma*I | CGTATCCCGGGGCTATGCGGAGACATACATTACCTAATCATC |
| F2- *Nhe*I | GAAATTTAGCTAGCTATGATGATGGGCTTCAACAG |
| R2- *SnaB*I | GAATAAGTACGTAGAGTGCGTATTACCCATTGAC |
| F3- *SnaB*I | GCATTTACGTACTTATTCATATTCATATACTAGTCC |
| R3- *Nhe*I | TAGCTGCTAGCTAAATTTCTGCTAAATCCAGATTTCAC |
| Snf-β-1F-*Xho*I | ATACAAAATAACTAAATTACGTAGCTAGCCTCGAGATGGGTAATACGCACTCGAC |
| Snf-β-1R-*Xho*I | CAGGTTTCTCATCTTTCCCTGTCTGCCTCGAGTTAGTTTTTGGGAAATACAGG |
| 2F | ATGGGTAATACGCACTCG |
| 2R | TTCTTGTACTTGCCGAATCC |
| 3F | GATAAGCATAAACCAGATCTGC |
| 3R | GTATCTGACATAGTCGAGCTTG |
| F4 | CCTCGTTCGATAATTACAGG |
| R4 | GATATGTGACAGATTTGCTACC |
| R5 | GCATATCACATTAGATCCATAGATC |
| Snf-β-F | CACCGCCTCAAGTTTATTGTG |
| Snf-β-R | CGGTCGACAAATCGTTATCTCC |
| 18SqPCR-F | GTCTTAGATGAGGTGGCCTGG |
| 18SqPCR-R | TGGCTAGAAACTATAAACAACCG |

* Restriction enzyme sites (6 bp nucleotides) are underlined.
